# Photogrammetry-Based Head Digitization for Rapid and Accurate Localization of EEG Electrodes and MEG Fiducial Markers Using a Single Digital SLR Camera

**DOI:** 10.3389/fnins.2017.00264

**Published:** 2017-05-16

**Authors:** Tommy Clausner, Sarang S. Dalal, Maité Crespo-García

**Affiliations:** ^1^Zukunftskolleg and Department of Psychology, University of KonstanzKonstanz, Germany; ^2^Center of Functionally Integrative Neuroscience, Aarhus UniversityAarhus, Denmark

**Keywords:** photogrammetry, 3D models, EEG, MEG, coregistration, electrode position, *janus3D*

## Abstract

The performance of EEG source reconstruction has benefited from the increasing use of advanced head modeling techniques that take advantage of MRI together with the precise positions of the recording electrodes. The prevailing technique for registering EEG electrode coordinates involves electromagnetic digitization. However, the procedure adds several minutes to experiment preparation and typical digitizers may not be accurate enough for optimal source reconstruction performance (Dalal et al., 2014). Here, we present a rapid, accurate, and cost-effective alternative method to register EEG electrode positions, using a single digital SLR camera, photogrammetry software, and computer vision techniques implemented in our open-source toolbox, *janus3D*. Our approach uses photogrammetry to construct 3D models from multiple photographs of the participant's head wearing the EEG electrode cap. Electrodes are detected automatically or semi-automatically using a template. The rigid facial features from these photo-based models are then surface-matched to MRI-based head reconstructions to facilitate coregistration to MRI space. This method yields a final electrode coregistration error of 0.8 mm, while a standard technique using an electromagnetic digitizer yielded an error of 6.1 mm. The technique furthermore reduces preparation time, and could be extended to a multi-camera array, which would make the procedure virtually instantaneous. In addition to EEG, the technique could likewise capture the position of the fiducial markers used in magnetoencephalography systems to register head position.

## Introduction

Brain source reconstruction of EEG scalp potentials has benefited from the increasing use of advanced head modeling techniques. In addition, combining the use of MRIs together with precise positioning and coregistration of recorded electrodes has increased source reconstruction performance. The localization of deep brain sources may especially benefit from accurate electrode determination because it affects the solution of the inverse problem, eminently when the signal to noise ratio (SNR) is low (Wang and Gotman, 2001; Koessler et al., 2008). Several methods exist for registering sensor positions, including manual measurement approaches, based on electromagnetic digitization, infrared, MRI, ultrasound, and photogrammetry (Le et al., 1998; Koessler et al., 2007; Zhang et al., 2014). However, final electrode determination accuracy varies widely across those methods. As Adjamian et al. (2004) demonstrated, fiducial-based coregistration, relying solely on anatomical landmarks produces remarkable displacements on final alignments of electrodes. Beltrachini et al. (2011) contended that deviations of electrode positions of less than approximately 5 mm result in negligible dipole source localization error. Another aspect to consider in quantifying source reconstruction performance is the resulting source SNR under realistic conditions of low sensor SNR. With increasing agreement of the true source configuration with the head model (which is heavily influenced by sensor coregistration accuracy), source SNR greatly improves, effectively lowering the detection threshold for weak sources (Dalal et al., 2014). Laboratory protocols must also take practical considerations into account. Factors important in designing EEG lab protocols often include preparation time, spatial and practical demands, as well as equipment cost and operational complexity (Russell et al., 2005; Koessler et al., 2011; Qian and Sheng, 2011; Reis and Lochmann, 2015). Routinely scanning volunteers with MRI with an EEG cap in place, despite its high accuracy, is not practical for many research labs depending on laboratory proximity, scanner availability, and potential scanning costs. Common electromagnetic digitizers, in turn, may not have sufficient accuracy for optimal performance (Dalal et al., 2014; Vema Krishna Murthy et al., 2014).

Photogrammetry using ordinary consumer-grade digital cameras can provide a cost-effective and accurate solution, and has already been used for a variety of applications in fields such as geomorphology or archaeology. For example, it has been used to create height maps of landscapes (Javernick et al., 2014), digitize cultural artifacts and monuments (McCarthy, 2014), and create cinematic effects like “bullet-time,” originally featured in *The Matrix* (1999). Existing applications related to neurophysiology include the localization of intracranial EEG electrode arrays in neurosurgery patients (Dalal et al., 2008). However, to the best of our knowledge, there are no low-cost, easy-to-use solutions employing photogrammetry-based scalp EEG electrode localization in practice. Qian and Sheng (2011) reported a proof-of-concept using a single SLR camera to determine EEG electrode positions by installing two planar mirrors forming an angle of 51.4°. A limitation of this approach is its high dependence on the precise mirror configuration and relative displacement of the measured head, and its use with human participants has not yet been reported. By using a similar procedure but employing a swivel camera over a head model, Baysal and Şengül (2010) demonstrated a rapid and accurate localization of sensor positions. However, electrode positions were again simulated using colored circles on an even scalp surface. The actual detection of electrodes on a real subject wearing an EEG cap has not been previously demonstrated. In 2005, Russell et al. proposed a new photogrammetry-based technique featuring 11 cameras mounted on a dome-shaped structure that is able to simultaneously capture images from different view angles around the participant's head. Although this system provides highly accurate EEG sensor positions, the proprietary software limits its use to a specific kind of EEG cap, and requires significant time to complete since the experimenter must manually select each electrode on the respective images.

Here, we present a rapid, accurate, and low-cost alternative method to register EEG electrode positions, using a single digital SLR camera and computer vision techniques implemented in our open-source toolbox, *janus3D*. Our method is based on photogrammetry, which has been demonstrated to provide highly accurate results with low-cost digital cameras (Baysal and Şengül, 2010; Qian and Sheng, 2011). Based on 2D DSLR camera images, 3D head models of subjects wearing an EEG cap are generated employing structure-from-motion (SfM) photogrammetry software. Electrode positions of a replica head model are determined using the photogrammetry-based approach and a common electromagnetic digitizer. Finally, electrode position accuracy and coregistration accuracy are analyzed and compared. Additionally, we introduce *janus3D*, a new MATLAB-based open source toolbox. This software was implemented as a GUI to allow the determination of highly accurate EEG sensor positions from the individual 3D-photogrammetry head models. Furthermore, it includes coregistration algorithms to align the models with their corresponding individual MRIs, as well as automatic template-based electrode labeling.

## Methods and materials

To evaluate the accuracy of our novel approach, we applied the method to a 3D printed full-scale replica head model of an adult subject wearing a 68-electrode EEG cap (Sands Research Inc., El Paso, TX, USA), as described in Dalal et al. (2014). The 3D-printed replica head was created after digitizing the subject's head with a high-resolution 3D laser scanner employing fringe projection (FaceSCAN^3D^, 3D-Shape GmbH, Erlangen, Germany). This device has a measurement uncertainty of 0.1 mm. The obtained mesh was 3D-printed and the replica head was scanned a second time to generate a mesh without the imperfections caused by the printing process (e.g., offset due to the thickness of the 3D printing filament used). On this mesh, two researchers independently determined the electrode positions in 3D software. The two sets of electrode positions were averaged and used as “ground truth” in the following analyses. A more detailed description on how the ground truth electrodes were obtained can be found in Dalal et al. (2014).

Our approach uses a single DSLR camera to capture 2D images that are necessary for the photogrammetry-based 3D reconstruction. Given that the replica head was printed in a uniform off-white color, but the reconstruction relies on color difference information, it was necessary to color the replica “cap.” The fabric of the replica EEG cap was subsequently colored similar to a real cap, also serving to provide sufficient contrast crucial for later texture-based automatic electrode detection.

**Figure 1:**
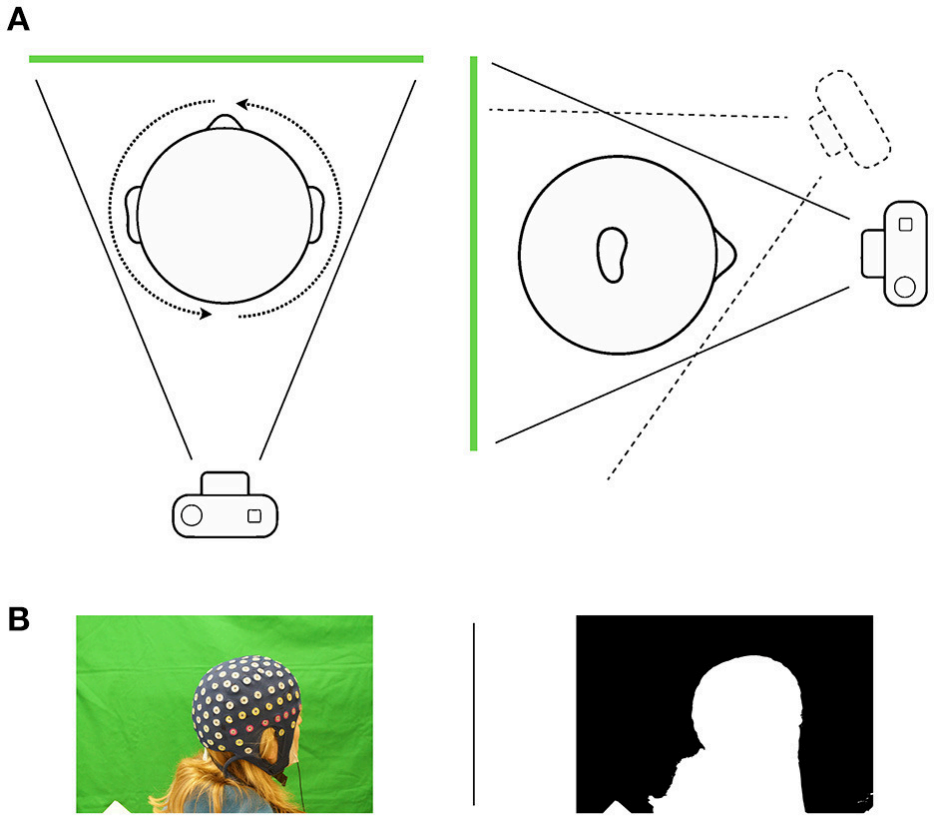
**(A)** Schematic illustration of the image capturing process. The subject moves in front of a chroma key greenscreen while sitting on a swivel chair. Capturing images from different height levels ensures coverage from the top of the head. **(B)** A picture—mask pairing as used by Agisoft PhotoScan. Setting areas to a value of 0 masks the background, whereas the object is defined by setting respective areas to a value of 1.

Fifty-six high-quality photographs of the replica head were captured using a 24-megapixel DSLR camera (Sony Alpha 65, Sony Corporation, Minato, Tokyo, Japan) equipped with a Sony DT 3.5–5.6/18–55 mm SAM II lens (35 mm focal length) mounted on a tripod. The exposure index was kept below ISO 800 to manage image noise. Aperture size was fixed at f/18 to avoid focal blur and maintain consistent optical properties across the photos. Motion blurring was reduced by firing the camera using a wired remote release. The replica head was placed in front of a 6' × 9' chroma key green screen backdrop fixed on the laboratory wall. Photos were taken by positioning the camera at 4 different height levels, in which the camera approximately described angles between 0° and 45° relative to the horizontal plane, in steps of about 15°. At each height level, the replica was rotated around its vertical axis in steps of about 20°–30° before taking a new photo. If necessary, the position of the tripod was slightly adjusted to fit the model into the camera's field of view. A schematic depiction of this procedure can be found in Figure 1A.

The reconstruction of the 3D mesh was performed using the commercial 3D photogrammetry software “PhotoScan” by Agisoft Agisoft LLC, St. Petersburg, Russia (2016). In general, any photogrammetry-based 3D reconstruction software can be used. However, PhotoScan was chosen because of its comfortable usability and fast reconstruction performance. It is able to compute 3D models based on the initial information provided by photographs and basic intrinsic features like focal length values, which are stored in the Exif metadata. Although prior camera calibration is recommended by the developer, it had little impact on the final results when the amount of pictures was sufficient (that is around 35 or more) and was therefore omitted from our final protocol. The implemented algorithm searches salient structures across all photographs and identifies matching points that are used to determine the camera position for each shot relative to the remaining.

To prevent faulty reconstructions and to reduce processing time and the amount of extraneous feature information, we masked irrelevant features in all photos (i.e., all information outside the object of interest) beforehand. For this purpose, we automatically created a binary image mask for each single picture, using an appropriate chroma key threshold. The respective threshold was selected automatically, but can be adjusted to increase contrast, which in the present case was not necessary. After importing all images into PhotoScan, they were coupled with their corresponding masks. Figure 1B depicts an example of a picture-mask pair for a human subject.

**Figure 2:**
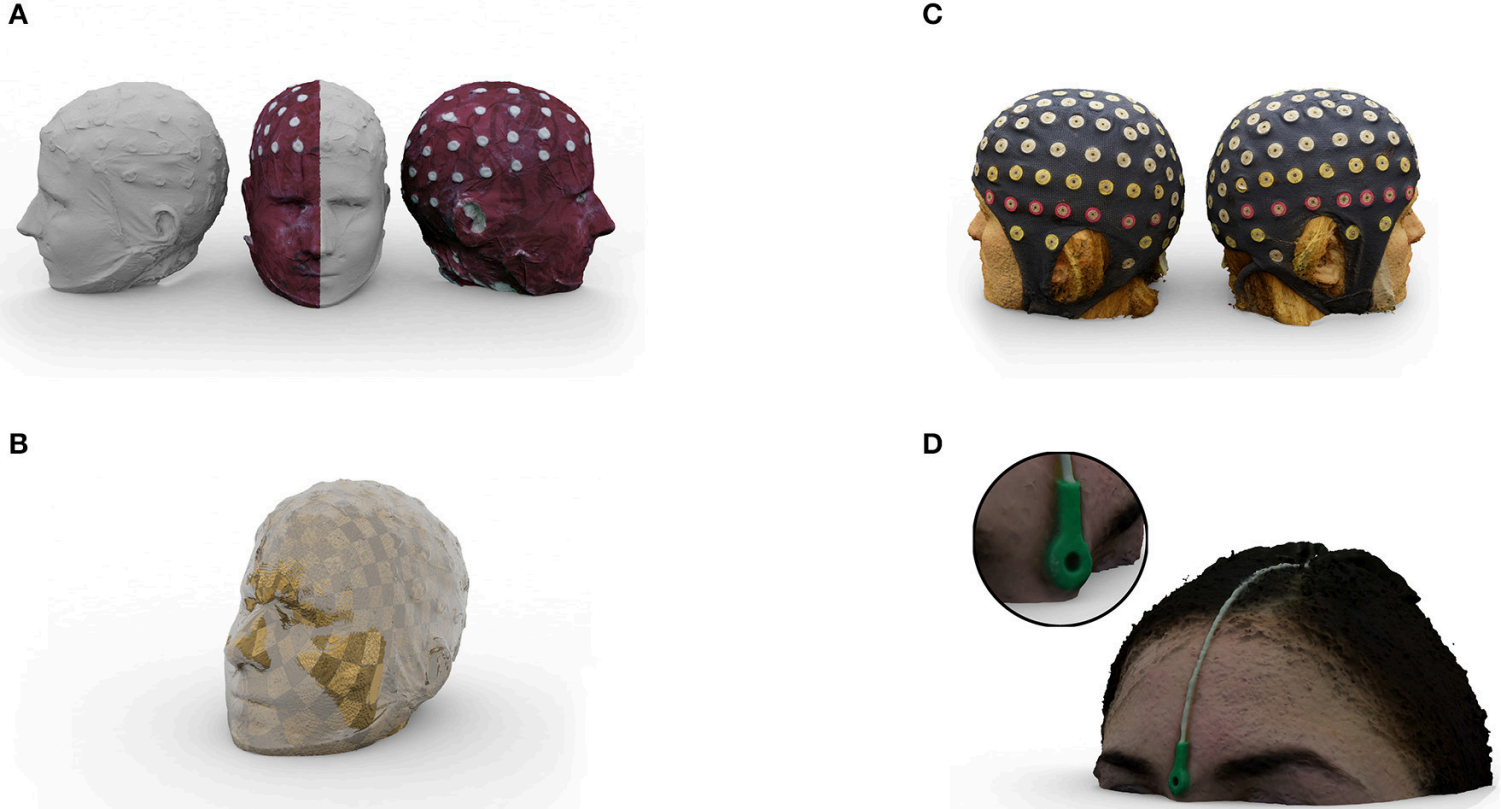
**(A)** Half textured 3D mesh of the replica head generated from 56 photographs. The white surface shows the untextured mesh and the dark red surface represents the texture we added. **(B)** Coregistration of the photogrammetry-based 3D reconstruction to structural MRI. The checkerboard surface was generated from the scalp surface of the segmented MRI and the glassy surface from the photogrammetry-based 3D reconstruction. Both models were coregistered using *janus3D*. **(C)** Example of a mesh obtained from a human subject wearing an ANT Waveguard 128 EEG cap. **(D)** Example of a mesh obtained from a human subject with an electrode attached to the nasion as commonly used for MEG head position measurement. The meshes were generated from 43 **(C)** or 55 **(D)** photographs, applying the same reconstruction procedure.

First, the algorithm creates a matching point cloud (MPC) and computes the corresponding set of camera positions based on that information. Afterwards, the MPC is densified by extracting additional points from corresponding high-resolution images in relation to each camera position. On the basis of this dense point cloud (DPC), PhotoScan generates a 3D polygonal mesh representing the object's surface. By following this procedure, we obtained a dense mesh of the replica's surface consisting of 1,717,422 faces and 859,513 vertices. Texture information was obtained by generic mapping after the geometry was computed. The final textured model was exported as Wavefront Object format (.obj) associated with a texture image file. Figure 2A depicts the model of the replica head resulting from the described procedure. For comparison, Figures 2C,D display examples of models obtained after applying the same procedure to human subjects wearing an ANT Waveguard 128 electrode cap or a facial MEG localization coil respectively.

**Figure 3:**
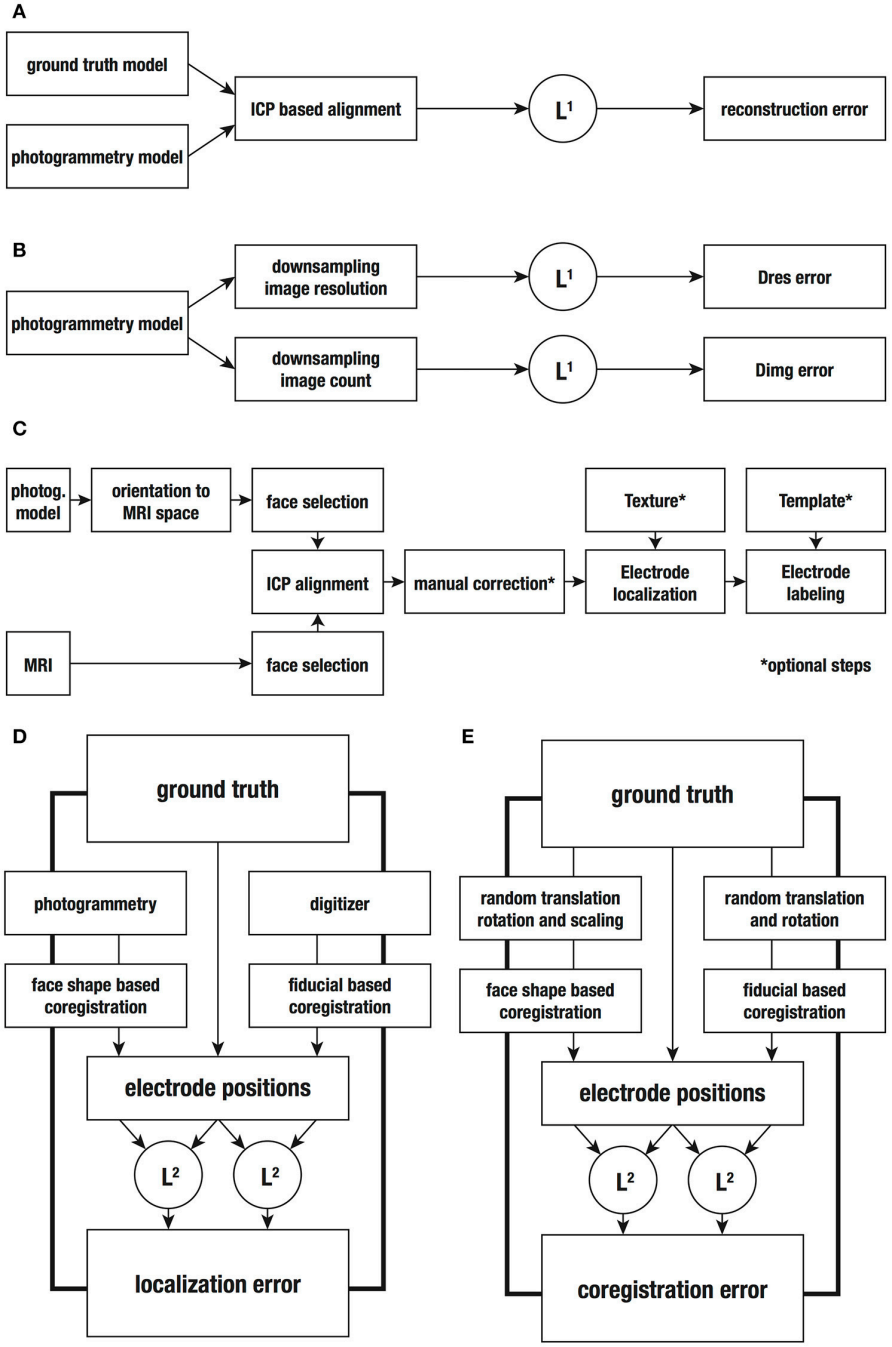
**Workflows followed for application and evaluation of the respective methods. (A)** Comparison between the ground truth and the photogrammetry-based 3D model to obtain the reconstruction error. **(B)** Evaluation of the downsampled models regarding image count (Dimg) or image resolution (Dres) by comparison with the ground truth model to obtain respective reconstruction errors. **(C)** Application workflow to obtain electrode positions from photogrammetry-based 3D models. **(D)** Determination of electrode positions with a digitizer and the photogrammetric method, and comparison with the ground truth positions to obtain the localization errors. **(E)** Procedure for evaluating the coregistration accuracy. ICP, Iterative closest point algorithm; L^1^, L^1^-norm to compute the offset of each vertex in each orthogonal direction; L^2^, L^2^-norm to compute Euclidean distance between vertex points.

To evaluate the quality of the obtained replica mesh, we applied an iterative closest point (ICP) algorithm (Besl and McKay, 1992) to the reconstructed 3D model generated by PhotoScan and the ground-truth 3D model obtained from the second scan of FaceSCAN^3D^. Before applying the ICP algorithm, the reconstructed 3D model is scaled using the same procedure as will be explained below when discussing MRI coregistration. After initial registration, the ICP algorithm attempts to minimize the sum of the squared distances for each point of the source point cloud to the closest point of the reference point cloud by a combination of translation and rotation, yielding a minimal distance solution. We evaluated the accuracy for each vertex of the reconstructed model, by localizing the closest vertex point in the ground-truth model and separately computed the offset in each orthogonal direction (L^1^-norm). A schematic depiction of this evaluation can be found in Figure 3A.

Further we studied the influence of the number and resolution of the pictures on the accuracy of the model reconstructed with PhotoScan (Figure 3B). For this purpose, we repeated the reconstruction procedure using downsampled sets of pictures where each factor was independently manipulated. To downsample image count (Dimg), we removed pictures in steps of 4, trying to keep a homogeneous coverage of the replica head. This resulted in 12 downsampled sets ranging from 56 images (full coverage) to 12 images, all with 24 megapixels. Image resolution (Dres) was downsampled using the same amount of 56 photographs. The resolution was reduced in software in steps of 4 megapixels, ranging from 24 to 8 megapixels. Additionally, we included in our analysis common image resolutions such as 4K, 1080p, and 720p, corresponding to 7.2, 1.75, and 0.78 megapixels respectively. This procedure resulted in 8 different sets of downsampled images. For later comparison, the downsampling rate was normalized by dividing the amount of pixels (image resolution × image count) by the highest value (24 megapixels × 56 pictures) and subtracting this value to 1. Hence, both 56 pictures at 12 megapixels resolution and 28 pictures at 24 megapixels correspond to 50% downsampling rate. Downsampling rates are indicated as values ranging from 0 (full information) to 1 (no information). Table 1 lists all downsampling steps including their respective downsampling rate. For each set of pictures, we registered the model resolution and the processing duration. All downsampled models, except those generated with image count of 12 and 16 pictures, were compared with the original one and the average mesh deviation was obtained.

**Table 1:** **Overview downsampling**.

**Image count**	**Megapixel**	**Dimg/Dres**	**Error in mm**
56	24	0.00	/
52	24	0.07	0.07
48	24	0.14	0.09
44	24	0.21	0.11
40	24	0.29	0.13
36	24	0.36	0.14
32	24	0.43	0.16
28	24	0.50	0.19
24	24	0.57	0.20
20	24	0.64	0.22
[16]	24	0.71	/
[12]	24	0.79	/
56	20	0.17	0.12
56	16	0.33	0.14
56	12	0.50	0.17
56	8	0.67	0.21
56	7.20	0.70	0.23
56	1.75	0.93	0.46
56	0.78	0.97	0.62

**Figure 4:**
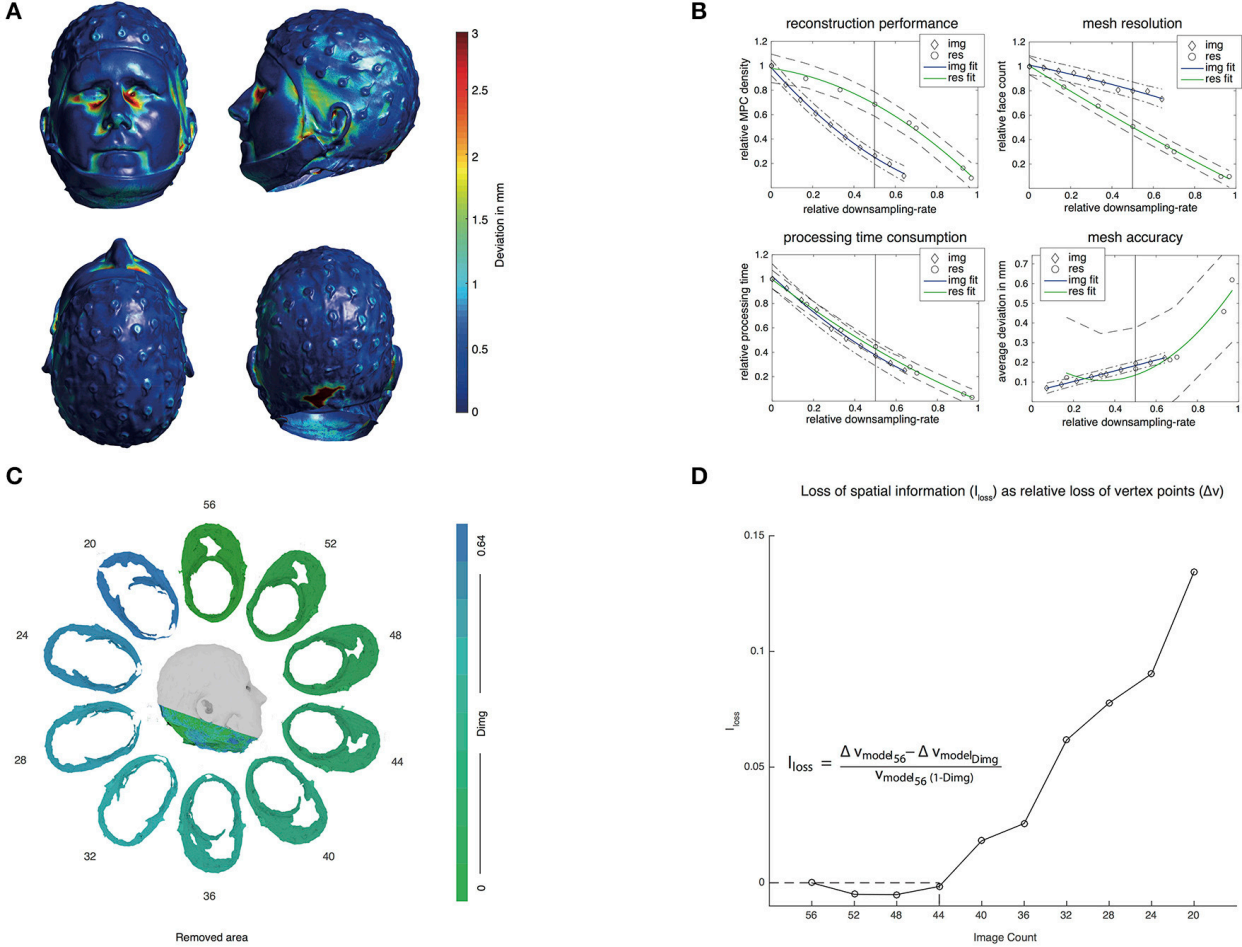
**(A)** 3D meshes indicating the distance to the closest vertex point from the reconstructed model to the ground truth model obtained from FaceSCAN^3D^. Difference values are plotted on the ground truth model. Vertex point distances are color coded, ranging from 0 mm to 2.95 mm (the 95^th^ percentile). **(B)** Descriptive curves for downsampling image count (Dimg) and image resolution (Dres). The blue and green lines indicate the quadratic fitting curves for the respective data. Dotted and dash-dotted lines indicate the respective 95% confidence interval of the fitting curves. X-axis values represent the relative downsampling rates corresponding to Table 1. Top left plots the MPC density relative to the full-resolution model, top right likewise for DPC density, bottom left for overall time consumption relative to the full-resolution model and bottom right for the average deviation to the full-resolution model in mm. **(C)** An example showing the area that needed to be removed in order to make the meshes covering a comparable area for each downsampling step for image count. **(D)** Additionally loss of vertex points in order to make the meshes covering a comparable area for each downsampling step (Dimg). The dashed area indicates the not interpretable result of having a negative loss of information as a function of downsampling. At 50% downsampling, 7.8% of spatial information was lost.

*Numbers in brackets indicate that a full model was not reconstructed. All other datapoints correspond to those in Figure 4B. Downsampling rates are given as values from 0 (full information) to 1 (no information) relative to the full-resolution model*.

Given that the reconstructed models showed slightly different mesh extensions, mismatching parts across meshes were removed to facilitate the computation of the average distance between the respective meshes after ICP based fine registration. Those extensions can occur at the outer boundary of the mesh because the respective 3D models generated with photogrammetry cannot be obtained in isolation. For example each set of images may contain different information from objects surrounding the object of interest. Given that the surrounding objects are not sampled completely (e.g., the surface on which the object of interest is placed) the reconstructed raw models may be slightly different at the borders that are in contact with other surfaces. Removed parts assured that all final meshes covered the same area from the original object (i.e., full head/face including electrodes). Thus, the removal did not influence the process itself, but made the meshes comparable. Note that our purpose was to measure errors regarding the reconstruction of the scanned object and not the overall scene. Figure 4C depicts the original full resolution model, surrounded by the respective part that was removed depending on Dimg.

Electrode position accuracy was obtained from the highest-resolution model by coregistering the 3D model to the individual MRI first and acquiring the respective electrode positions afterwards. Note that accurate coregistration is critical for acquiring accurate EEG electrode positions.

First, the 3D model was reoriented and rescaled from PhotoScan's arbitrary coordinate system into MRI space. Therefore, it was crucial to correctly select features that are shared by the reconstructed mesh and the rendered MRI. It has been proposed that face-to-face matching works best when selections include parts from below the nose to upper facial regions—excluding cheeks—as illustrated in previous studies (e.g., Kober et al., 2003; Koessler et al., 2011). Thus, parts around the nose bone (i.e., forehead, cheekbones and eyebrows) turned out to be optimal, due to their high rigidity. We used the point clouds of the selected facial segments to compute a scaling factor between the 3D-model and the MRI. For this purpose, we divided the mean L^2^-norm (i.e., the Euclidean distance) between each vertex point $v_{i}^{MRI}$ from the MRI segment and its centroid *C*^*MRI*^ by the mean Euclidean distance between each point $v_{i}^{model}$ from the 3D-model segment and its centroid *C*^*model*^, as indicated by the following equation:

$$s_{xyz}=\frac{\sum_{i\;=\;1}^{N_{MRI}}\|\left(v_{i}^{MRI}-C^{MRI}\right)\|/N^{MRI}}{\sum_{i\;=\;1}^{N_{model}}\|\left(v_{i}^{model}-C^{model})\right\|/N^{model}}$$

Each centroid was defined as the mean coordinate of all points within each segment, calculated across each dimension separately. Then, all points of the 3D model were multiplied by this scaling factor. Afterwards both segments were coregistered by applying an ICP algorithm. A rigid body transformation matrix transforming the facial selection of the model to the MRI was obtained. This transformation matrix was applied to the whole 3D model. An example of this step is shown in Figure 2B.

Identifying electrode shapes on the textured 3D model took place hereafter, by recognizing circular structures on the mesh surface from various view angles. This was achieved by adding a binarized version of the model's texture to the mesh. The binary texture was created by thresholding the model's texture to maximize the contrast difference between electrodes and cap. From 10 different perspectives, a 2D Hough transform (Yuen et al., 1990; Atherton and Kerbyson, 1999) for circular shape detection was performed, as implemented in the function “imfindcircles” from MATLAB's Image Processing toolbox. Hereby, multiple view angles can compensate for ellipsoid electrodes at occluding boundaries of the head. Those points were back-projected into 3D space yielding the final electrode positions. Five slightly displaced electrodes were manually corrected on a 3D representation of the textured mesh.

In our experience, the amount of electrodes that need manual adjustment is around 5% of all electrodes (depending on the contrast between electrodes and the surrounding texture). However, since electrode positions can be manually selected on a textured representation of the mesh, electrode selection can be done precisely due to instant visual feedback.

In addition, electrodes were labeled automatically, based on a majority vote. For this purpose, seven independent sets of template electrodes were used. Those were coregistered using two automatically detected landmark electrodes (Fpz and Oz) followed by an ICP affine registration. The respective label was selected according to the label of the closest distance of the electrode to be labeled to each of the sets of template electrodes. This yields seven proposed labels of which the one was chosen that would receive the most “votes,” leading to an inaccuracy of around 5%. However, for the automatic labeling algorithm to work properly, it is crucial that electrodes that need to be labeled and the respective sets of template electrodes are in accordance with respect to number and relative position of electrodes. This automatic labeling procedure was implemented and performed in *janus3D*.

Figure 3C depicts the full workflow used to coregister and obtain EEG electrode positions out of individual MRIs and 3D models.

We estimated the error committed during the determination of EEG electrode positions and compared it to the performance of an electromagnetic digitizer, ANT Neuro Xensor (ANT Neuro, Enschede, Netherlands). Using the stylus pen, two experienced experimenters registered electrode positions 3 times directly on the replica. One electrode (TP10) was poorly reproduced on the replica head and was therefore removed from further analyses. The remaining 67 electrode positions were coregistered to the individual MRI using NUTMEG (Dalal et al., 2011), based on common fiducial points (i.e., nasion and pre-auricular points). For each method, Euclidean distances between electrode positions and the ground truth positions were determined (L^2^-norm). Both methods were compared applying Wilcoxon's signed-rank tests to the respective deviations. Figure 3D gives an overview of the steps used to compare the accuracy of both methods.

The coregistration error was distinguished from the method-specific localization error. Note that to determine the electrode positions using the electromagnetic digitizer and the photogrammetry-based method, it is necessary to apply different coregistration approaches, which are based either on the fiducial points or matching of the facial surface, respectively. To compare the accuracy of both coregistration approaches, we repeated the coregistration on spatially shifted versions of the 3D model and the electrode positions determined previously, together with the fiducial points. The spatial shifts were achieved by applying random linear transformations, including rotations between 1° and 360° and translations between 1 and 100 mm for each orthogonal direction. Additionally, a random scaling factor between 1 and 5 was applied to the 3D model to transpose it into an arbitrary coordinate system, simulating the PhotoScan reconstruction. Next, we calculated the Euclidean distances between the original electrode positions and the electrode positions after coregistering the modified versions. Finally, we compared both sets of Euclidean distances using Wilcoxon's signed-rank tests to obtain an estimation of the coregistration error committed in both methods (see also Figure 3E).

Pure electrode position accuracy was evaluated by ICP aligning each set of electrodes to the ground truth electrode set and tabulating the residual error (i.e., Euclidean distance) for each electrode position. Subsequently, the performance of the coregistration methods were evaluated with respect to each other by applying Wilcoxon's signed rank test to these residual errors.

Electrode determination and MRI coregistration as described, were implemented and performed in *janus3D*. Furthermore, this toolbox includes image-processing functions to facilitate the creation of binary masks from the photos captured by the DLSR camera. *janus3D* allows importing 3D models in Wavefront OBJ format (Wavefront Technologies, Toronto, Canada) and MRIs in NifTI file format. The software automatically generates a 3D mesh derived from the MRI's scalp surface calling the Fieldtrip functions “ft_read_mri,” “ft_volumesegment,” and “ft_prepare_mesh” setting the method to “projectmesh” (Oostenveld et al., 2011).

**Figure 5:**
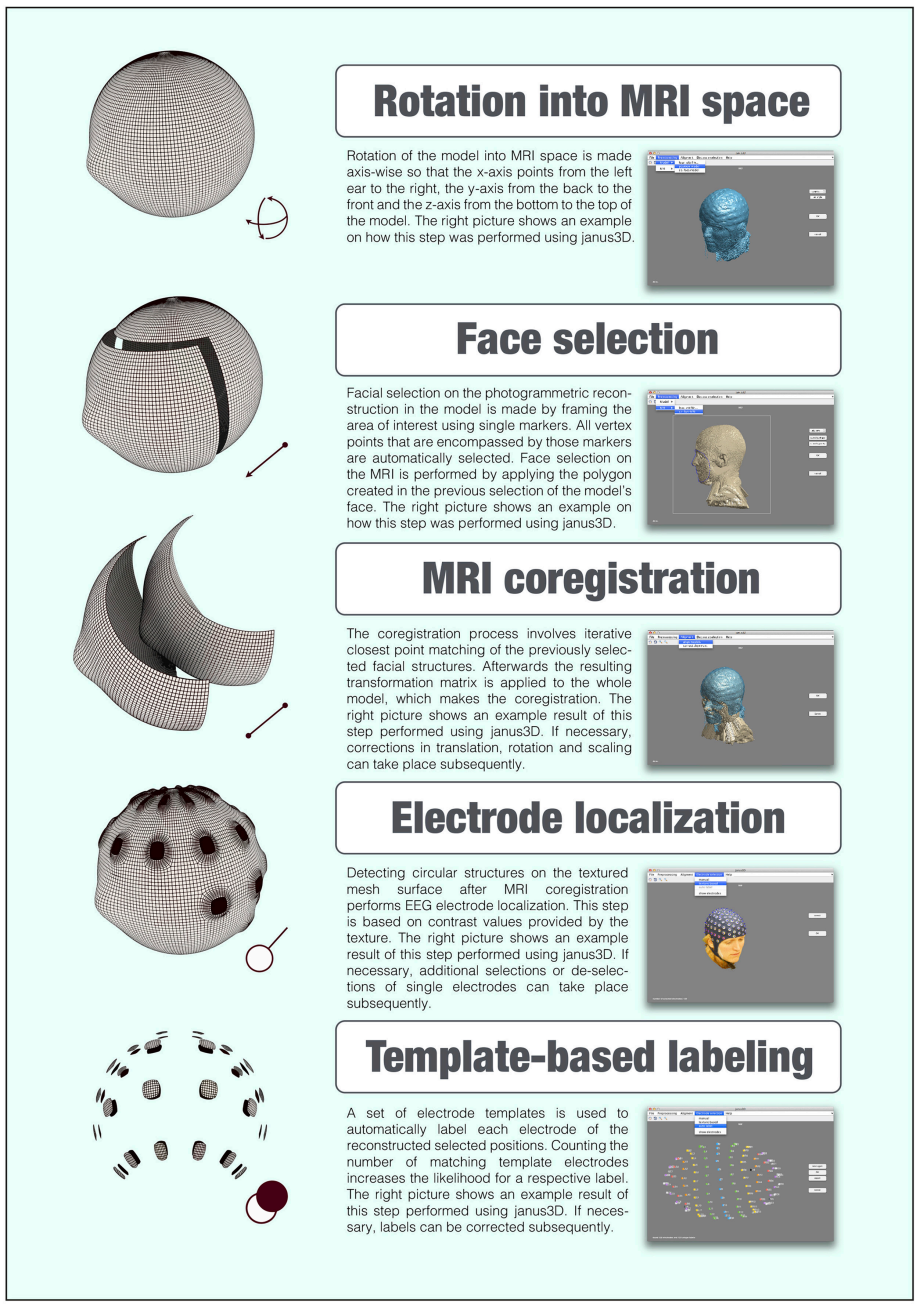
**Flowchart depicting the general *janus3D* work flow**.

A graphical user interface (GUI) is provided to allow the visualization and manipulation of the rendered MRI and reconstructed 3D model. After a manual pre-orientation into MRI space, it is possible to select similar facial sections in both meshes that will be coregistered employing an ICP algorithm. If necessary, manual corrections can take place hereafter, as the software provides functions for translation, rotation and scaling. Electrode determination and labeling are facilitated by comfortable GUI functions. The resulting electrode positions are provided as raw model positions and projected orthogonally onto the MRI's surface. For automatic labeling of arbitrary EEG cap layouts, *janus3D* includes an easy-to-use template builder. Figure 5 illustrates the workflow of the whole process, depicting example screenshots for each step. *janus3D* requires MATLAB 2015a including the Image Processing and Computer Vision System Toolboxes and Fieldtrip (Oostenveld et al., 2011). It is compatible with all platforms running MATLAB and is available as standalone application for Mac OSX and Linux. *janus3D* is available at https://janus3d.github.io/janus3D_toolbox/ under the MIT license.

## Results

We evaluated the accuracy of the 3D reconstruction by computing the minimal distance between each vertex point of the reconstructed model obtained from PhotoScan and the ground truth model obtained from FaceSCAN^3D^. The average distance across all vertex points was 0.90 mm (median: 0.52 mm; SD: 1.00 mm). 95% of all vertex points showed a deviation smaller than or equal to 2.95 mm. Figure 4A depicts this difference for each vertex point represented on the surface of the second scan using FaceSCAN^3D^.

The influence of image count and image resolution are depicted in Figure 4B (top left) for MPC density, (top right) the respective face count of the mesh, (bottom left) the over-all processing time consumption and (bottom right) the average deviation relative to the model with highest image count and resolution. MPC density reduction was more pronounced for Dimg than for Dres, whereas DPC density reduction was more pronounced for Dres. Although a 50% downsampling in both cases meant the same total amount of pixels contributing to the reconstruction, the MPC was 2.6 times denser in the Dres condition than in Dimg (Figure 4, top left panel) whereas the final mesh resolution, expressed by the face count, was 1.6 times higher for Dimg compared to Dres (Figure 4A, top right panel). The overall reduction in processing time was similar for Dimg and Dres. The mesh accuracy diminished with increasing Dimg downsampling, although at a low rate, reaching a maximal deviation of 0.22 mm. Dres showed remarkably low accuracy in the last two downsampling steps: at 1080p and 720p resolution, the deviation was of 0.46 and 0.62 mm, respectively. When the resolution was kept above 4K (equivalent to 7.2 MP), mesh accuracy was only influenced slightly, culminating at 0.23 mm. A detailed overview of error values related to the actual downsampling rates can be found in Table 1.

**Figure 6:**
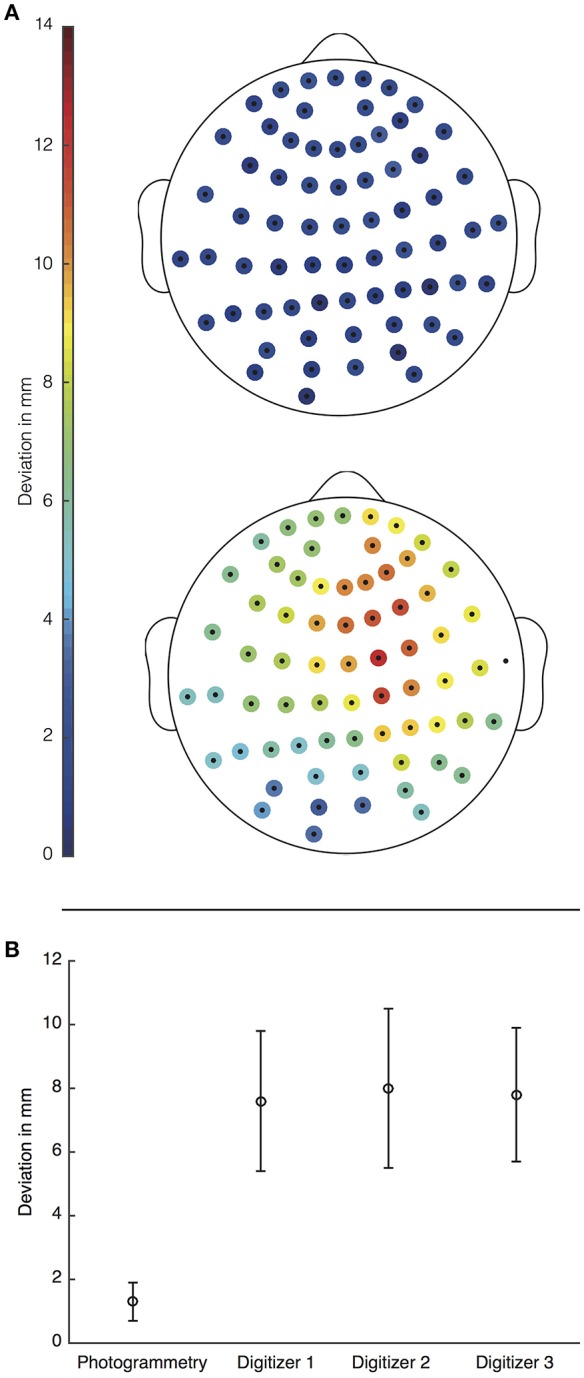
**(A)** Topographies for deviations of all electrode coordinates in mm. Those determined with the photogrammetry-based approach (top) had a mean deviation of 1.3 mm whereas the deviation obtained from the electromagnetic digitizer (bottom) was 7.6 mm. **(B)** The average deviation (SD) for both methods was 1.3 mm (0.6 mm) for the photogrammetry (**A**—top) and 7.6 mm (2.2 mm) (**A**—bottom), 8.0 mm (2.5 mm) and 7.8 mm (2.1 mm) for the electromagnetic digitizer. Error bars indicate the respective standard deviation.

The mean (SD) difference between electrode positions determined using the photogrammetry-based approach and the ground truth was 1.3 mm (0.6 mm). Electrode positions obtained using the ANT Xensor^TM^ electromagnetic digitizer unveiled a mean difference of 7.8 mm across the 3 measurements (mean [SD]: 7.6 mm [2.2 mm], 8.0 mm [2.5 mm], 7.8 mm [2.1 mm]). Indeed, Wilcoxon's signed rank test revealed that electrode positions determined with the photogrammetry-based approach had significantly smaller errors than those measured with the electromagnetic digitizer (*p* < 10^−4^, for all 3 measurements). Figure 6A depicts the deviation for each single electrode of the respective method relative to the ground truth electrodes (top) for the photogrammetry-based approach and (bottom) for the first measurement using the electromagnetic digitizer.

We also evaluated the accuracy of the coregistration methods used for each approach on spatially shifted versions of the electrode positions. The mean (SD) deviation of the new electrode positions compared with the original ones was 0.78 mm (0.24 mm) and 6.14 mm (0.65 mm) after coregistration based, on facial surface matching and the fiducials, respectively. Wilcoxon's signed rank test revealed that errors of electrode positions due to coregistration were significantly smaller for surface matching compared to the fiducial-based method (*p* < 10^−4^).

ICP aligned pure electrode positions differed over all 3 measurements (mean [SD]: 1.48 mm [0.76 mm], 1.58 mm [0.91 mm], 1.37 mm [0.68 mm]). Wilcoxon's signed rank test revealed a significant (*p* < 0.01) difference to the model's deviation that was 1.00 mm (0.54 mm) for all 3 comparisons.

## Discussion

Compared to the ground truth model, the photogrammetry-based 3D reconstruction deviates 0.52 mm (median) over all vertex points. This error is partially setup-dependent because both the amount and the resolution of the pictures that are used for generating the model can influence the reconstruction performance. Although a resolution of 7.2 megapixels yields negligibly small deviations of 0.23 mm compared to the full-resolution model, the deviation at 1080p (0.78 megapixels) and 720p (1.75 megapixels) increases up to 0.62 mm. Despite the increased error rate at the lowest resolution is relatively small, the final 3D meshes appear slightly smeared, due to the significantly lower resolution of the models. Downsampling image count caused negligible effects on the model's error. Nevertheless, the matching point algorithm was affected by image count. In fact, a reduction in the detected matching points observed at the highest downsampling rates (16 and 12 pictures) strongly impaired the reconstruction, making it impossible to obtain complete models. These results suggest that obtaining a complete model reconstruction will require at least 20 different camera perspectives. Furthermore, Figure 4D depicts the relative loss of information. The amount of vertices that additionally needed to be removed from the highest resolution model to make all models covering the same area increased noticeable when less than 40 images were used for the reconstruction. This means that the loss of information was higher than the expected loss due to downsampling itself. It is therefore advisable to acquire more than 32 images to keep additional information loss below 5%.

Independent from that, the final mesh resolution increases with increasing resolution of the camera used. Differences in electrode localization performance can be assumed as of the same range that the whole model would expose after downsampling. Since the vertex points of each electrode are drawn from the same set of vertex points used for comparing model reconstruction performance, only a systematic bias specifically toward electrode vertices, could have had potential influence to the final electrode position. Therefore electrode position accuracy in dependency of downsampling was not tested separately as it was assumed being directly linked to the overall mesh reconstruction error.

In the present study only DSLR cameras were tested. Conclusions on how other types of cameras would perform (e.g., compact cameras) cannot be drawn. Lens aberrations and inconsistencies could affect reconstruction quality, but prior camera calibration may compensate for these effects and allow the use of lower-end cameras. In our study, we did not use prior camera calibration because, in our experience, this step mainly improves the 3D reconstruction only under weak light conditions or when too few images were captured. Agisoft also recommends the use of prior camera calibration if images of different cameras are merged in a single set.

Electrode localization accuracy benefits from the relatively small 3D reconstruction error associated with the photogrammetry-based approach. It outperforms common electromagnetic digitizers (see also Figure 6). As ANT states on their webpage, the technical inaccuracy of this electromagnetic digitizer is less than 2 mm ?. This is still a relatively high inaccuracy compared to the median deviation of the photogrammetry-based approach found here (0.52 mm), which may even be an overestimation. Remondino et al. (2014) compared different 3D reconstruction approaches for different kinds of objects. For static head models reconstructed using Agisoft PhotoScan they observed a measurement inaccuracy of 0.1 mm, which is even smaller than what we found.

The high technical accuracy of the photogrammetry-based approach is reflected on the accuracy of the electrode positions. Whereas a standard electromagnetic digitizer had a mean error of 7.8 mm, the photogrammetry-based approach only deviated by 1.3 mm. These findings are in line with previous work (e.g., Baysal and Şengül, 2010; Dalal et al., 2014). The small errors observed across electrodes (*p* < 10^−4^) suggest that our novel approach may significantly enhance the accuracy of EEG source reconstruction (e.g., Khosla et al., 1999; Michel et al., 2004; Dalal et al., 2014).

Our analyses also show that an important part of the accuracy gain is due to smaller MRI coregistration errors. Whereas electromagnetic digitizers commonly use a fiducial-based coregistration (mean error 6.1 mm), our photogrammetric approach is based on a coregistration involving facial surface matching (mean error 0.8 mm), which is significantly more accurate (*p* < 10^−4^). Fiducial-based coregistration only relies on a few points that are manually defined on the subject and on the MRI volume. On the other hand, coregistration based on facial surface matching can use several thousand points that are matched iteratively by an ICP algorithm. Nevertheless, facial sections selected from the rendered MRI and the 3D model should include the same facial region; otherwise the iterative alignment can fail.

Taking this point into account, *janus3D* was designed to make this step as easy and reliable as possible. It features a facial selection that is based on the boundary of the first face selection, which can be performed either on the MRI or the reconstructed 3D model. The boundary shape is used as an overlay template for the corresponding second selection. Additionally, electrode detection also benefits from automatized algorithms implemented in *janus3D*. The software is able to automatically determine electrode positions using texture-based shape detection, only occasionally requiring manual correction. Even then, this procedure is faster and more reliable than the single electrode selection with an electromagnetic digitizer because the user is able to determine electrode positions on a static mesh. Direct visual feedback allows the user to detect and instantly correct inaccurate selections.

Nevertheless, there are some limitations that need to be considered. As the photogrammetry-based approach relies on proper image quality, a well-illuminated environment is necessary when acquiring the photos. To avoid image noise, ISO values of the camera should be kept below ISO 800 and the aperture size should be f/8 or lower. Depending on the camera, in our experience, standard ceiling lights in a typical laboratory do not provide sufficient light. However, the models depicted in Figures 2C,D were acquired using standard ceiling lights. This explains the somewhat rough appearance of the facial features in our reconstruction. Setting up additional lighting is not only beneficial, but also necessary in most indoor environments. Multiple lights or diffusers should be installed to avoid creating shadows that may “travel” across the head with rotation. Due to the nature of human skin, reflections should also be avoided as they similarly impact the reconstruction results. The replica model we used was less reflective than human skin. For that reason more than 20 pictures are likely to be required when scanning a human subject. In our experience, sufficient reconstruction results are obtained at a number above 35 pictures. Further testing also revealed that using 3 cameras close to each other overcomes most of the imperfections. Shadows and reflections are recognized at the same time from different view angles and therefore compensate for each other. Another benefit of this setup is that only 20 rotational steps are necessary, if the cameras are aligned such that two cameras face the front from two opposing perspectives and one camera faces the top of the head. An array of cameras would be an alternative implementation that would acquire all viewpoints simultaneously, avoiding the need for rotation, and would likely further improve the measurement accuracy. Any facial movements or movements due to the subject's rotation would be eliminated and shadow information and reflections would serve as a feature instead of a possible source for inaccuracies. Furthermore, it would speed up image acquisition to just a few seconds. Our results imply that more than 20 cameras would be needed, with a corresponding increase in equipment costs.

Finally, 3D-model based MRI coregistration could similarly improve MEG coil coregistration as Vema Krishna Murthy et al. (2014) showed by employing a Microsoft Kinect camera. Source reconstruction performance tested on a phantom head increased by 137% using Kinect 3D coregistration compared to a Polhemus electromagnetic digitizer. Since the Kinect camera yielded an average coregistration error of 2.2 mm, we would expect improvements of MEG source reconstruction performance using our novel approach on a similar scale. To achieve this, MEG reference coils would need to be referred to facial landmarks as those used for registering the head of the subject to the MEG's coordinate system. A possible solution could be the use of visible markers on the face of the subject that later could be found on the textured mesh.

## Conclusion

Single DSLR camera photogrammetry serves as a rapid method for accurate EEG electrode detection. Additionally it is a cost-effective alternative to common methods like electromagnetic digitizers and outperforms them in measurement and MRI coregistration accuracy. Finally, reconstructed 3D models of subjects wearing an EEG cap, created with a common DSLR camera and photogrammetry software may improve the results of ultimate beamformer solutions, when conducting source analysis (Dalal et al., 2014).

## Author contributions

Idea and conception: SD, MC; development of methodological and analytical strategies: TC, SD, MC; data acquisition: TC, MC; data analysis and programming of *janus3D*: TC; Drafting the manuscript: TC; Critical revision: TC, SD, MC.

## Funding

This work was supported by the Zukunftskolleg of the University of Konstanz (SD and MC), the Deutsche Forschungsgemeinschaft (grant DA1485-1/1 to SD), ERA-Net NEURON via the Bundesministerium für Bildung und Forschung (BMBF grant 01EW1307 to SD), and the European Research Council (Starting Grant 640488 to SD).

### Conflict of interest statement

The authors declare that the research was conducted in the absence of any commercial or financial relationships that could be construed as a potential conflict of interest.
